# The dopamine D1–D2DR complex in the rat spinal cord promotes neuropathic pain by increasing neuronal excitability after chronic constriction injury

**DOI:** 10.1038/s12276-021-00563-5

**Published:** 2021-02-09

**Authors:** Yi-Ni Bao, Wen-Ling Dai, Ji-Fa Fan, Bin Ma, Shan-Shan Li, Wan-Li Zhao, Bo-Yang Yu, Ji-Hua Liu

**Affiliations:** 1 Jiangsu Key Laboratory of TCM Evaluation and Translational Research, School of Traditional Chinese Pharmacy, China Pharmaceutical University, Nanjing, Jiangsu 211198 China; 2 State Key Laboratory of Natural Medicines, School of Traditional Chinese Pharmacy, China Pharmaceutical University, Nanjing, Jiangsu 210009 China

**Keywords:** Molecular neuroscience, Cellular neuroscience

## Abstract

Dopamine D1 receptor (D1DR) and D2 receptor (D2DR) are closely associated with pain modulation, but their exact effects on neuropathic pain and the underlying mechanisms remain to be identified. Our research revealed that intrathecal administration of D1DR and D2DR antagonists inhibited D1–D2DR complex formation and ameliorated mechanical and thermal hypersensitivity in chronic constriction injury (CCI) rats. The D1–D2DR complex was formed in the rat spinal cord, and the antinociceptive effects of D1DR and D2DR antagonists could be reversed by D1DR, D2DR, and D1–D2DR agonists. Gαq, PLC, and IP3 inhibitors also alleviated CCI-induced neuropathic pain. D1DR, D2DR, and D1–D2DR complex agonists all increased the intracellular calcium concentration in primary cultured spinal neurons, and this increase could be reversed by D1DR, D2DR antagonists and Gαq, IP3, PLC inhibitors. D1DR and D2DR antagonists significantly reduced the expression of p-PKC γ, p-CaMKII, p-CREB, and p-MAPKs. *Levo*-corydalmine (*l*-CDL), a monomeric compound in *Corydalis yanhusuo* W.T. Wang, was found to obviously suppress the formation of the spinal D1–D2DR complex to alleviate neuropathic pain in CCI rats and to decrease the intracellular calcium concentration in spinal neurons. *l-*CDL-induced inhibition of p-PKC γ, p-MAPKs, p-CREB, and p-CaMKII was also reversed by D1DR, D2DR, and D1–D2DR complex agonists. In conclusion, these results indicate that D1DR and D2DR form a complex and in turn couple with the Gαq protein to increase neuronal excitability via PKC γ, CaMKII, MAPK, and CREB signaling in the spinal cords of CCI rats; thus, they may serve as potential drug targets for neuropathic pain therapy.

## Chronic pain: Cutting communication for nerve pain signals

Insights into dopamine-mediated neuronal activity in the spinal cord may lead to more effective relief for some chronic pain patients. Up to 10% of adults worldwide live with some form of pain due to nerve damage (neuropathy), and few treatment options are available. The neurotransmitter dopamine is thought to regulate pain-related signaling in the spinal cord, and researchers led by Ji-Hua Liu and Bo-Yang Yu of China Pharmaceutical University, Nanjing, have identified a way to interfere with this signaling. Working with a rat neuropathic pain model, the researchers focused on a protein complex that acts as a receptor for dopamine in spinal neurons. Once activated, this complex initiates cellular processes that mediate the pain response. Chemical agents that block receptor assembly counter these processes, highlighting a promising mechanism to target in neuropathic pain patients.

## Introduction

Neuropathic pain results from damage to or disease that affects the somatosensory system^1^. Population-based estimates of the incidence of neuropathic pain in adults range from 7 to 10% worldwide, and the current treatments for neuropathic pain have limited therapeutic efficacy in patients^2^.

Hyperactivity of spinal cord sensory neurons plays an essential role in the process of neuropathic pain^3^. The spinal cord is the first relay site in the transmission of nociceptive information in the central nervous system (CNS)^4^. When an injury occurs, the activated presynaptic neurons release numerous excitatory neurotransmitters, such as glutamate (Glu) and the neuromodulator substance P (SP), which bind to specific receptors on the postsynaptic membrane, such as AMPA, NMDA, and neurokinin 1 (NK1) receptors, thus leading to the activation of spinal neurons^5^. Stimulated membrane-bound receptors trigger a massive influx of intracellular calcium and activate a series of intracellular calcium-dependent downstream signal transduction pathways, including the calcium-calmodulin dependent kinase II (CaMKII), protein kinase C γ (PKC γ) and downstream mitogen-activated protein kinase (MAPK) pathways. MAPKs, including extracellular signal-regulated kinase (ERK), p38 MAPK, and c-Jun NH2-terminal kinase (JNK), can promote the activation of transcription factors, such as NF-κB and cAMP response element-binding protein (CREB) to further activate spinal neurons and enhance pain transmission^6^.

The detailed roles of dopamine D1 receptor (D1DR) and D2 receptor (D2DR) in pain and their mechanisms still need to be fully elucidated, and the effects of these receptors in chronic neuropathic pain remain largely unexplored. Descending dopaminergic pathways have been reported to contribute to pain modulation^7,8^. Supraspinal and spinal dopamine receptors might have distinct effects on pain modulation^9^. The exact effects of spinal dopamine receptors on chronic neuropathic pain need to be further explored. D1DR and D2DR have been reported to be mainly expressed in neurons in the spinal cord^10^. Activated spinal neurons play an important role in the development and maintenance of chronic pain^11^. Inhibiting the activation of spinal neurons can markedly attenuate neuropathic pain. D1DR couples to the Gαs/olf protein to activate cyclic adenosine monophosphate (cAMP), and D2DR couples to the Gαi/o protein to inhibit adenyl cyclase (AC)^12^. Activation of cAMP signaling can lead to the phosphorylation of cAMP response element-binding protein (CREB) to increase the excitability of rodent hippocampal neurons and striatal neurons, while inhibition of cAMP decreases neuronal excitability^13^. The dopamine D1–D2DR heteromer was first identified in the rat striatum^14^ and has been reported to couple to the Gαq protein, a finding that suggests the existence of a direct link between dopamine and calcium signaling^15–17^. Increases in the intracellular calcium concentration have been implicated in increases in the excitability of neurons^18^ and the development of chronic pain.

We hypothesized that D1DR and D2DR form a complex in the spinal cord, leading to Gαq-dependent intracellular calcium mobilization and thus increasing neuronal excitability to facilitate the generation and development of neuropathic pain. Furthermore, we explored the potential of *levo*-corydalmine (*l-*CDL) as a safe and effective compound that can regulate these receptors.

## Materials and methods

### Ethics statement

The study was executed in strict compliance with the stipulations of the International Association for the Study of Pain and under protocols approved by the Animal Experimentation Ethics Committee of China Pharmaceutical University.

### Drugs and reagents

Primary antibodies for Western blotting, i.e., anti-phospho-PKC γ, anti-phospho-CREB, anti-phospho-CaMKII, and anti-phospho-MAPK, were purchased from Cell Signaling Technology (Beverly, MA). Poly-L-lysine (PLL), an anti-glyceraldehyde 3-phosphate dehydrogenase antibody (GAPDH), and Fluo-3/AM were purchased from Sigma-Aldrich (St. Louis, MO). Secondary antibodies for immunofluorescence were obtained from Jackson ImmunoResearch Laboratories Inc. (PA). Anti-D1DR and anti-D2DR antibodies were obtained from Santa Cruz Biotechnology (Santa Cruz, CA), and an anti-D1DR antibody was purchased from Abcam (Cambridge, MA). Neurobasal medium and fetal bovine serum (FBS) were both obtained from Gibco (Gaithersburg, MD). Soybean trypsin and trypsin inhibitors were obtained from Atlanta Biologicals (Norcross, GA). Agonists and antagonists were purchased from Tocris Bioscience (Ellisville, MO), NHS magnetic beads were purchased from Enriching Biotechnology (Nanjing, China), and all other reagents used for Western blotting, including Trizma base (Tris base), sodium dodecyl sulfate (SDS), methylenebis-acrylamide (ACR), ammonium persulfate (APS) and tetramethylethylenediamine (TEMED), were purchased from Sigma-Aldrich (St. Louis, MO).

D1DR (NM_012546)- and D2DR (NM_012547)-specific small interfering RNAs (siRNAs) were synthesized by GenePharma Co. (Shanghai, China). The sequences of the control siRNAs (con siRNAs) were random rearrangements of the nucleotide sequences of the D1DR siRNAs and D2DR siRNAs. The sequences of the D1DR siRNAs were as follows: A, 5′-GGUGACCAACUUCUUUGUCTT-3′, and B, 5′-GACAAAGAAGUUGGUCACCTT-3′. The sequences of the D2DR siRNAs were as follows: A, 5′-CUACUAUGCCAUGCUGCUCTT-3′ and B, 5′-GAGCAGCAUGGCAUAGUAGTT-3′.

### Animals

A total of 370 male Sprague-Dawley rats (180–220 g) supplied by the Experimental Animal Center of Yangzhou University (Jiangsu, China) were used in this study. The rats were raised in groups of three per cage on a 12-h light/dark cycle at 22–25 °C and provided free access to food and water. The animals used for the experiments were randomly assigned to different groups.

### Primary culture of spinal neurons

As previously described, primary spinal neurons were cultured^19^. Briefly, when the animals reached breeding age, they were housed together for breeding in a harem breeding system (i.e., two females per male) in the afternoon or evening^20^. After three nights, the animals were separated based on sex. Pregnancy was confirmed by weight gain specifically in the lower torso and the presence of small bumps. Pregnant animals were euthanized on E13 (E0 = first day following the mating day) in a CO_2_ chamber according to institutional guidelines, and the embryos were removed carefully from the uterus under aseptic conditions^21^. The embryos were removed and immediately placed in cold buffer. The spinal cords of the embryos were dissected, and the meninges were carefully removed. Then, the spinal cords were incubated at 37 °C for 25 min in 0.15% trypsin, washed and centrifuged at 200 × *g* at room temperature (RT) for 4 min. Then, the tissues were resuspended in solution containing DNase and soybean trypsin inhibitor. The supernatant was collected and centrifuged (4 min, 200 × *g*) at RT to obtain cells 15 min later. The cells were plated in 35-mm confocal dishes (precoated with poly-L-lysine (PLL, 0.1 mg/mL)) at a density of 2.5 × 10^5^ cells/mL in neurobasal plating medium comprising 2% NS21, 5% FBS, 1% HEPES and 0.5% *l*-glutamine (Gibco, Gaithersburg, MD, USA). The cultured cells were incubated in 5% CO_2_ at 95% relative humidity at 37 °C. The neurobasal plating medium was completely replaced with neurobasal growth medium comprising 2% NS21, 1% HEPES and 1% *l*-glutamine after 24 h, and the neurobasal growth medium was replaced every 3 days.

### Chronic constriction injury (CCI) of the sciatic nerve

The model was established according to the experimental method of Bennett and Xie^22^. The rats were abdominally injected with 50 mg/kg body weight sodium pentobarbital. Under anesthesia, the left sciatic nerve of each rat was exposed and separated at the mid-thigh level. The sciatic nerve was tied with four ligatures (4–0 chromic gut sutures) spaced 1 mm apart. 4–0 chromic gut sutures were used to suture the muscle, and wound clips were used to close the incision. Penicillin (4000 units) was intramuscularly injected into each rat.

### Intrathecal injection procedure

The rats were placed in the prone position, and the midpoint between the tips of the iliac crest was located. Intrathecal injections into the spinal cord between the L5–L6 lumbar vertebrae were performed manually with a glass microsyringe with a 30-gauge needle. Successful puncture was confirmed by a tail flick in the absence of changes to baseline responses. Each rat received a volume of 20 μL.

### Behavioral tests

The mechanical withdrawal threshold (MWT) and thermal withdrawal latency (TWL) were assessed on two successive days before and 1, 3, 5, 7, 9, 11, 13, and 14 days after CCI. Testing was performed during daylight hours from 08:00 to 18:00 h. All experiments were conducted in a blinded manner.

The MWT was tested with von Frey filaments (Woodland Hills, Los Angeles). The rats were acclimated to the testing apparatus for 15 min, and the responses of the paws to mechanical stimuli were assessed. The left hind paw of each rat was stimulated from below with a von Frey filament (1.4–15 g) for 3–5 s until the rat appeared to lick and/or raise its foot. The stimuli were presented in 5 trials separated by 5-min intervals, and the average threshold was calculated.

The TWL was tested using a plantar analgesia meter (model 37370; Ugo Basile Biological Instruments). The rats were placed in a plexiglass box and allowed to habituate for 15 min. The intensity of the stimulating light source was set to 45 °C, and the latency to a behavioral response was recorded for each rat. The maximum stimulation time was set to 15 s, and the test was repeated 5 times at 5-min intervals.

### Western blotting

L4–L6 spinal cord tissues were collected and homogenized in RIPA lysis buffer containing a protease inhibitor cocktail and phosphatase inhibitors. Equal amounts of protein were loaded and separated on 10–15% SDS polyacrylamide gels. Then, the proteins were transferred to polyvinylidene difluoride membranes. The membranes were blocked in TBST containing 5% BSA or 5% skim milk for 2 h at RT. Then, the membranes were incubated with primary antibodies against GAPDH, p-PKC γ, p-JNK, p-ERK, p-p38, p-CREB, and p-CaMKII overnight at 4 °C. After being washed in 0.1% Tris-buffered saline Tween (TBST), the membranes were incubated with a corresponding HRP-conjugated anti-mouse or anti-rabbit antibody for 2 h at RT. Quantity One-4.6.5 software (Bio-Rad Laboratories) was used to analyze the data.

### Coimmunoprecipitation

A D1DR antibody or D2DR antibody (10 μg) was added to NHS-activated magnetic beads (Enriching Biotechnology, Jiangsu, China) and incubated in 500 μL coupling buffer at RT for 4 h. Then, the supernatant was gently removed, 500 μL of blocking buffer was added, and the mixture was incubated at 4 °C for 1 h. Rat tissues (L4–L6 spinal cord) were lysed with RIPA buffer (10 mM NaF, 30 mM HEPES, 150 mM NaCl, 1% Triton, and 0.01% SDS), added to the bead-Ab complex and incubated for 4 h at RT. After that, the immunoprecipitates were collected, washed with wash buffer for 30 s (3–5 times), and incubated for 5 min at RT with elution buffer (100 μL). The supernatant was collected, SDS sample buffer was added, and the mixture was incubated for 6 min at 100 °C. Then, the proteins were loaded and separated on 10–15% SDS polyacrylamide gels, and the proteins of interest were measured by Western blotting.

### Calcium imaging

Fluo-3/AM staining and an LSM 700 inverted confocal laser scanning microscope (Carl Zeiss, Germany) were used to measure intracellular calcium concentrations. Primary spinal cord neurons were loaded with Fluo-3/AM (5 μM) in Locke’s buffer (in mM: 2.3 CaCl_2_, 154 NaCl, 8.6 HEPES, 1.0 MgCl_2_, 5.6 KCl, 0.1 glycine and 5.6 glucose, pH 7.4) containing 5 μg/mL BSA in the dark at 37 °C for 50 min. Then, the cells were washed three times with Locke’s buffer and incubated in the dark for another 15 min. Fluorescence at 488 nm excitation was assessed with an LSM 700 microscope (Carl Zeiss, Germany).

### Cell culture and transfection

The human D1DR (NM_000794.5) and D2DR (NM_000795.4) genes were synthesized by General Biosystems (Chuzhou, China) and cloned into the pCitrine-N1 and pmCherry-N1 plasmids at the XhoI/EcoRI sites to yield the recombinant plasmids pD1DR-Citrine-N1 and pD2DR-mCherry-N1. The procedure used to insert the pEYFP-N1 vector into the pCitrine-N1 vector was described previously^23^. HEK293 cells were cultured in DMEM comprising 10% FBS at 37 °C in 5% CO_2_. Transient transfection of HEK293 cells was performed with Invitrogen™ Lipofectamine™ 2000 Transfection Reagent according to the manufacturer’s instructions. One day before transfection, HEK293 cells were plated in 35-mm confocal dishes and transfected with 3 μg of DNA/dish. The ratio of DNA coding for receptors labeled with Citrine to DNA coding for receptors labeled with mCherry was 1:1 (w/w). The cells were transfected for at least 24 h.

### Microscopy of cells

After transfection, the cells were stimulated with 30 μM SKF 38393, quinpirole, or SKF 83959 for 30 min (pretreatment with 10 μM SCH-23390, L-741,626 or *l*-CDL for 15 min). An LSM 700 inverted confocal laser scanning microscope (Carl Zeiss, Germany) was used to obtain fluorescence microscope images. The Citrine fluorescent protein was excited with a 488 nm laser line, and the mCherry fluorescent protein was excited with a 555 nm laser line. Emission was recorded between 495 and 588 nm (Citrine) and between 578 and 700 nm (mCherry). Only cells showing coexpression of D1DR and D2DR were selected for analysis. For colocalization analysis of pD1DR-Citrine-N1 and pD2DR-mCherry-N1, ImageJ software was utilized. The degree of colocalization of the receptors was estimated by Pearson’s correlation coefficient (PCC).

### Statistical analysis

All data are expressed as the mean ± SEM. SPSS Rel 15 (SPSS Inc., Chicago, IL, USA) was utilized for the statistical analyses. The data were statistically evaluated by one-way analysis of variance (ANOVA) or two-way ANOVA followed by Bonferroni’s post hoc tests, and significance was set at *P* < 0.05.

## Results

### Antagonizing spinal D1DR and D2DR inhibited mechanical allodynia and thermal hypersensitivity in CCI rats

**Fig. 1:**
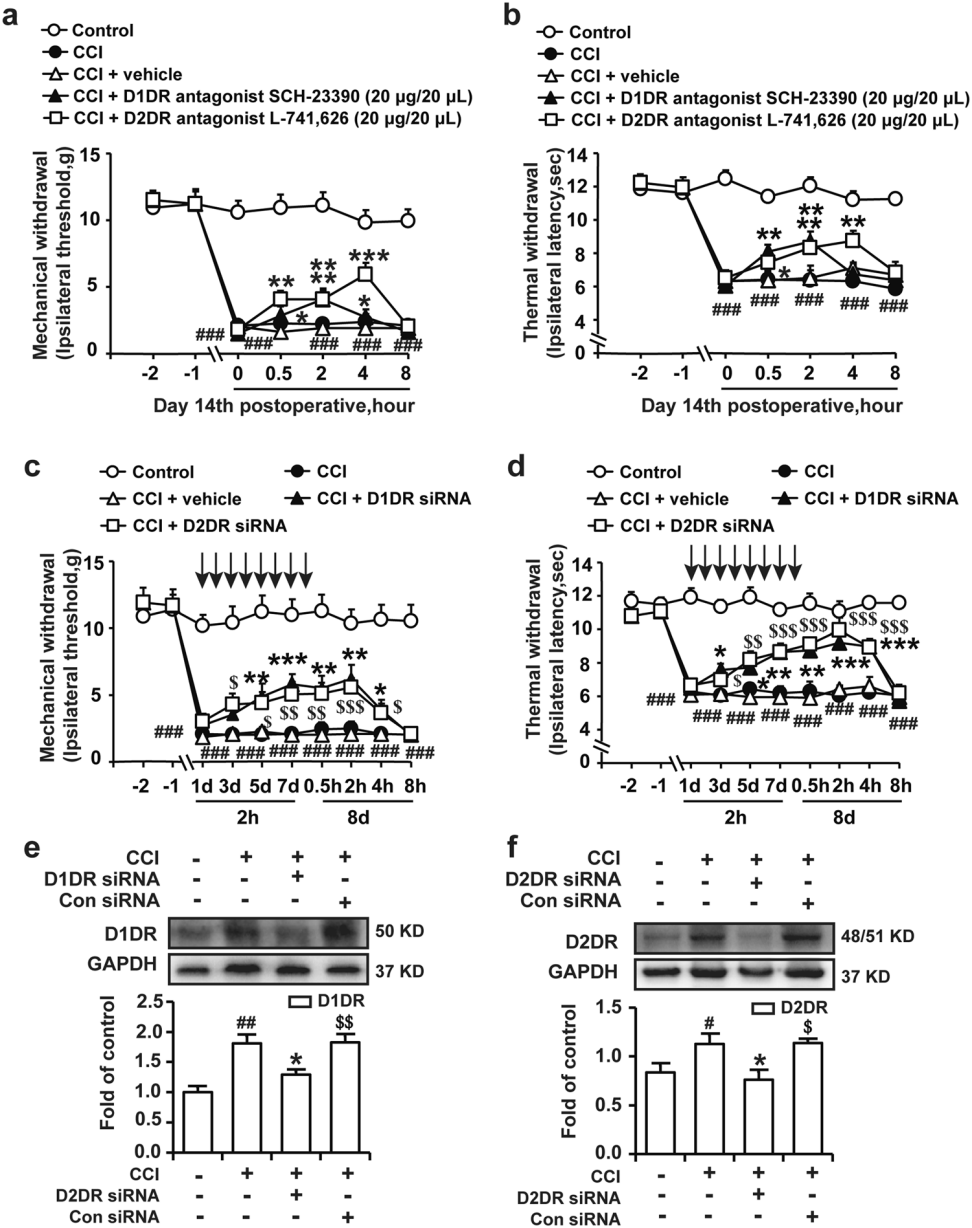
Antagonizing spinal D1DR and D2DR inhibited mechanical allodynia and thermal hypersensitivity in CCI rats. **a**, **b** The time course of the MWTs and TWLs of CCI rats after a single administration of the D1DR antagonist SCH-23390 (20 μg/20 μL, i.t.) and D2DR antagonist L-741,626 (20 μg/20 μL, i.t.). **c**, **d** The time course of the MWTs and TWLs of rats with CCI after multiple daily intrathecal injections of D1DR and D2DR siRNA/con siRNA for 8 consecutive days (*n* = 6; ^###^*P* < 0.001, compared with the control group; *P < 0.05, **P < 0.01, ***P < 0.001, compared with 0 h; ^$^*P* < 0.05, ^$$^*P* < 0.01, ^$$$^*P* < 0.001, compared with 0 h). **e**, **f** The expression of D1DR and D2DR in CCI rats after intrathecal administration of D1DR and D2DR siRNA (1 μg/20 μL, i.t.) for 8 days (*n* = 6; ^###^*P* < 0.001 vs the control group; *P < 0.05, **P < 0.01, ***P < 0.001 vs the CCI group; ^$^*P* < 0.05, ^$$^*P* < 0.01, ^$$$^*P* < 0.001 vs the siRNA group).

The D1DR antagonist SCH-23390 and the D2DR antagonist L-741,626 were intrathecally administered on postoperative day 14 to assess their analgesic effects in CCI rats. As shown in Fig. 1a, b, the behavioral tests revealed that SCH-23390 (20 μg/20 μL, i.t.) and L-741,626 (20 μg/20 μL, i.t.) significantly ameliorated mechanical and thermal hypersensitivity. Compared to that of vehicle or control siRNA con siRNA (1 μg/20 μL), intrathecal administration of a D1DR-targeting siRNA or D2DR-targeting siRNA (1 μg/20 μL) daily for 8 consecutive days beginning on postoperative day 14 dramatically reduced D1DR protein or D2DR protein expression (Fig. 1e, f). Furthermore, intrathecal administration of D1DR siRNA and D2DR siRNA dramatically alleviated mechanical and thermal hyperalgesia induced by CCI in rats (Fig. 1c, d).

### Spinal D1DR and D2DR formed a complex in CCI rats and increased the intracellular calcium concentration in spinal neurons through the Gαq-PLC-IP3 pathway

**Fig. 2:**
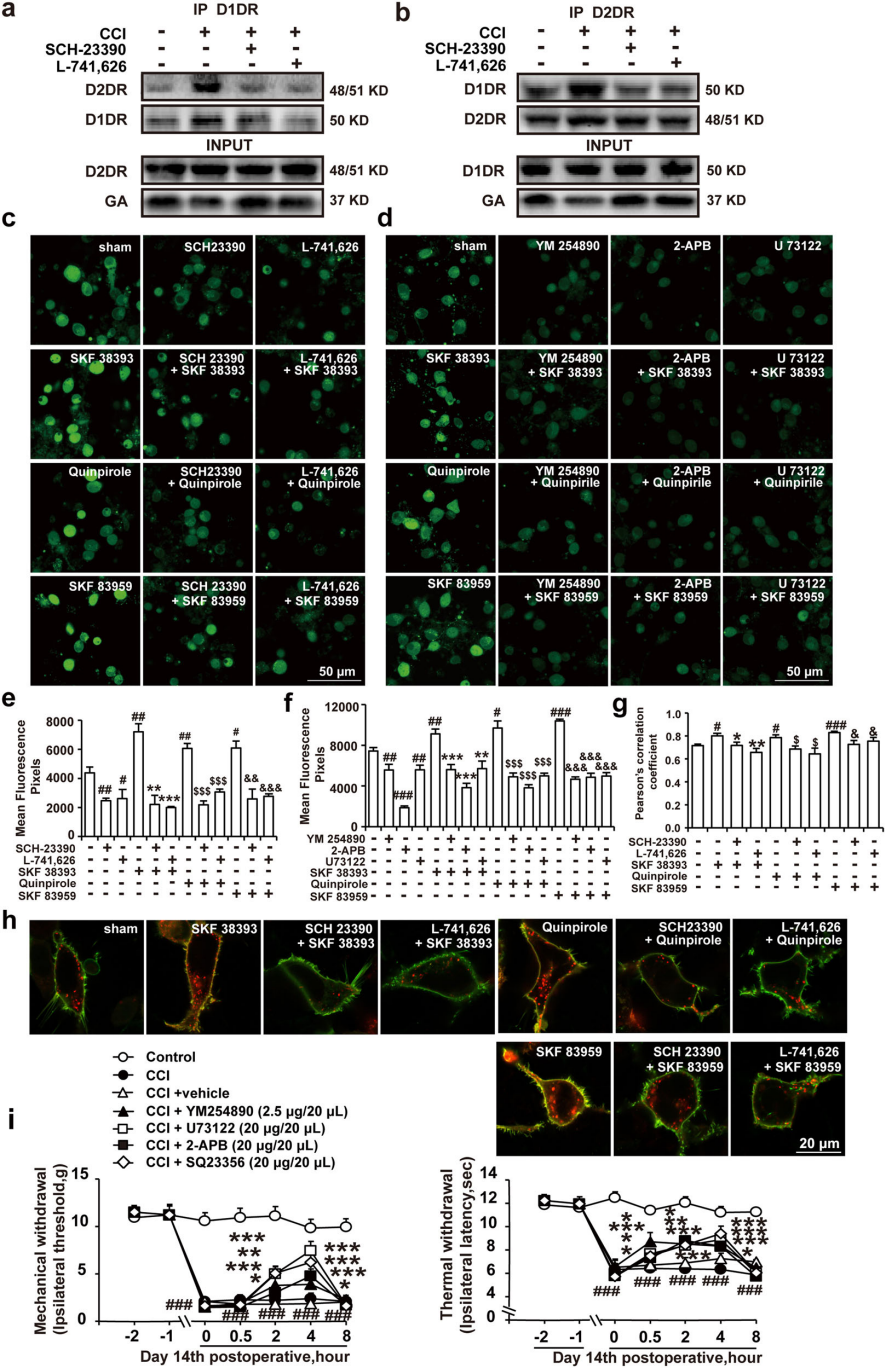
Spinal D1DR and D2DR formed a complex in CCI rats and increased intracellular calcium concentrations in spinal neurons through the Gαq-PLC-IP3 pathway. **a**, **b** Coimmunoprecipitation of D1DR and D2DR in the spinal cords of CCI rats after intrathecal administration of D1DR and D2DR antagonists (20 μg/20 μL, i.t.). **c**, **e** Immunofluorescence showing the calcium concentration after administration of SCH-23390 (10 μM) and L-741,626 (10 μM) and the effect of SCH-23390 (10 μM) and L-741,626 (10 μM) on SKF 38393 (30 μM), quinpirole (30 μM), and SKF 83959 (30 μM)-induced increases in the calcium concentration in spinal neurons (*n* = 4; ^#^*P* < 0.5, ^##^*P* < 0.01 vs the control group; **P < 0.01, ***P < 0.001 vs the SKF 38393 group; ^$$$^*P* < 0.001 vs the quinpirole group; ^&&^*P* < 0. 01, ^&&&^*P* < 0.001 vs the SKF 83959 group). **d**, **f** Immunofluorescence showing the calcium concentration after administration of YM254890 (10 μM), 2-APB (10 μM) and U73122 (10 μM) and the effect of YM254890 (10 μM), 2-APB (10 μM) and U73122 (10 μM) on SKF 38393 (30 μM), quinpirole (30 μM), and SKF 83959 (30 μM)-induced increases in the calcium concentration in spinal neurons. **h**, **g** Immunofluorescence showing the colocalization of D1DR and D2DR after administration of SCH-23390 (10 μM) and L-741,626 (10 μM) and the effect of these drugs on SKF 38393 (30 μM), quinpirole (30 μM), and SKF 83959 (30 μM)-induced increases in colocalization in the HEK293 cell membrane (*n* = 4; ^#^*P* < 0.05, ^##^*P* < 0.01, ^###^*P* < 0.001 vs the control group; *P < 0.05, **P < 0.01, ***P < 0.001 vs the SKF 38393 group; ^$^*P* < 0.05, ^$$^*P* < 0.01, ^$$$^*P* < 0.001 vs the quinpirole group; ^&&&^*P* < 0.001 vs the SKF 83959 group). **i** The time course of the MWTs and TWLs of rats with CCI after a single administration of YM254890 (2.5 μg/20 μL, i.t.), U73122 (20 μg/20 μL, i.t.), 2-APB (20 μg/20 μL, i.t.) and Q23356 (20 μg/20 μL, i.t.) (*n* = 6; ^###^*P* < 0.001 vs the control group; ***P < 0.001 vs the CCI group).

It has been reported that D1–D2DR complex activation generates a novel Gαq-PLC-IP3-mediated calcium signal that leads to increased neuronal excitability^24^, and increased neuronal excitability greatly contributes to chronic pain^25^. D1–D2DR complex-mediated signaling has been found to be inhibited by both D1DR and D2DR antagonists^15^. The results showed that D1DR and D2DR could form a D1–D2DR complex in the spinal cord after CCI, and both SCH-23390 (20 μg/20 μL, i.t.) and L-741,626 (20 μg/20 μL, i.t.) inhibited D1–D2DR complex formation (Fig. 2a, b). Fluorescence intensity can be regarded as an indicator of the cytoplasmic calcium concentration^26^. The results of in vitro studies in primary cultured spinal neurons indicated that compared with the control, the D1DR agonist SKF 38393, D2DR agonist quinpirole and D1–D2DR complex agonist SKF 83959 (all 30 μM) evoked a rapid elevation of calcium fluorescence (Fig. 2c–f) that was markedly suppressed by treatment with 10 μM L-741,626 and SCH-23390 (Fig. 2c, e) and the Gαq, PLC, and IP3 inhibitors YM254890, U73122, and 2-APB (Fig. 2d, f). Treatment with SCH-23390, L-741,626, YM254890, U73122, or 2-APB alone effectively reduced the intracellular calcium concentration in primary spinal cord neurons. In addition, we performed cell transfection experiments to verify the colocalization of D1DR and D2DR in the HEK293 cell membrane. Pearson coefficient correlation (PCC) analysis of fluorescence colocalization showed that the colocalization of D1DR and D2DR was evidently higher in cells treated with SKF 38393, quinpirole, and SKF 83959 (all 30 μM) than in control cells and that the colocalization degree was markedly decreased by treatment with 10 μM L-741,626 and 10 μM SCH-23390 (Fig. 2g, h). The behavioral results showed that YM254890, U73122, and 2-APB obviously alleviated CCI-induced neuropathic pain (Fig. 2i). As D1DR activates the AC-cAMP-PKA cascade, it also increases neuronal excitability^27^, thus promoting the development of chronic pain. The inhibitor SQ23356 was found to relieve CCI-induced neuropathic pain (Fig. 2i).

### The antinociceptive effects of D1DR and D2DR antagonists were abolished by pretreatment with D1DR, D2DR, and D1–D2DR complex agonists in CCI rats

**Fig. 3:**
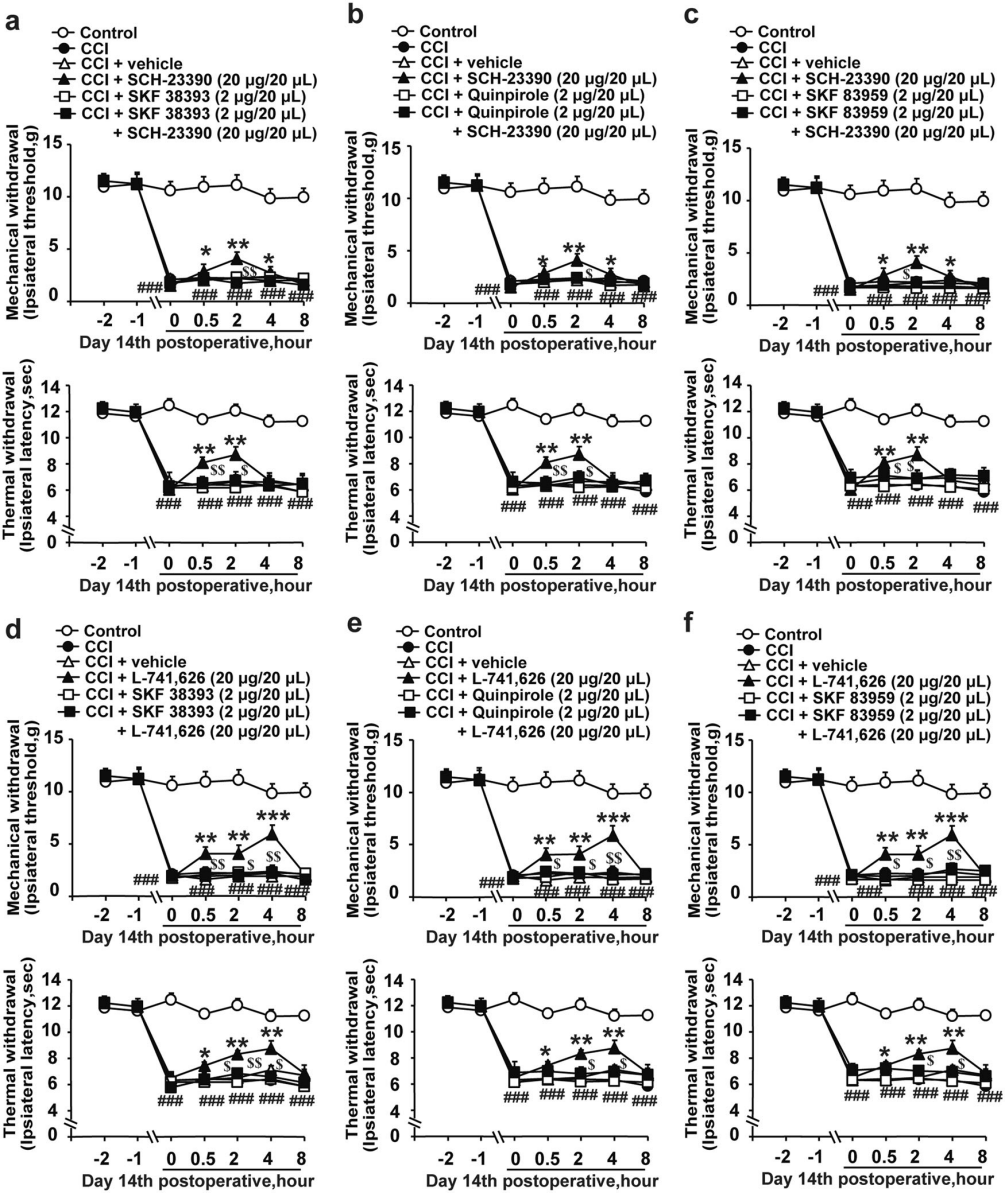
The antinociceptive effects of D1DR and D2DR antagonists were abolished by pretreatment with D1DR, D2DR, and D1–D2DR complex agonists in CCI rats. **a**–**c** The time course of the MWTs and TWLs of CCI rats after coadministration of the D1DR agonist SKF 38393 (2 μg/20 μL, i.t.), D2DR agonist quinpirole (2 μg/20 μL, i.t.), and D1–D2DR heteromer agonist SKF 83959 (2 μg/20 μL, i.t.) with the D1DR antagonist SCH-23390 (20 μg/20 μL, i.t.). **d**–**f** The time course of the MWTs and TWLs of rats with CCI after coadministration of the D1DR agonist SKF 38393 (2 μg/20 μL, i.t.), D2DR agonist quinpirole (2 μg/20 μL, i.t.), and D1–D2DR heteromer agonist SKF 83959 (2 μg/20 μL, i.t.) with the D2DR antagonist L-741,626 (20 μg/20 μL, i.t.) (*n* = 6; ^###^*P* < 0.001 vs the control group; *P < 0.05, **P < 0. 01, ***P < 0.001 vs the CCI group; ^$^*P* < 0.05, ^$$^*P* < 0.01 vs the D1DR and D2DR antagonist group).

It has been suggested that the activation of D1DR and D2DR by subtype-specific agonists promotes the formation of D1–D2DR heteromers^15,28^. D1DR and D2DR antagonists can also attenuate D1–D2 heteromer-mediated signaling^29,30^. To verify whether D1DR and D2DR antagonist-induced antinociception was mediated by the D1–D2DR complex, D1DR, D2DR, and D1–D2DR complex agonists were used. Intrathecal administration of the D1DR agonist SKF 38393, the D2DR agonist quinpirole and the D1–D2DR complex SKF 83959 (2 μg/20 μL, i.t., 15 min before SCH-23390 and L-741,626 treatment) abolished the SCH-23390 (Fig. 3a–c) and L-741,626 (Fig. 3d–f)-induced inhibition of mechanical and thermal pain sensitivity in CCI rats. As inhibition of AC also alleviated CCI-induced neuropathic pain, the D1DR agonist SKF 83822 (which robustly stimulates AC and affects calcium release) was used. SKF 83822 (2 μg/20 μL, i.t.) had no influence on the antinociceptive effects of SCH-23390 and L-741,626, as shown in the Supplemental Materials (Fig. s1a, b).

### Antagonizing spinal D1DR and D2DR inhibited the activation of PKC γ, CaMKII, MAPK, and CREB in CCI rats

**Fig. 4:**
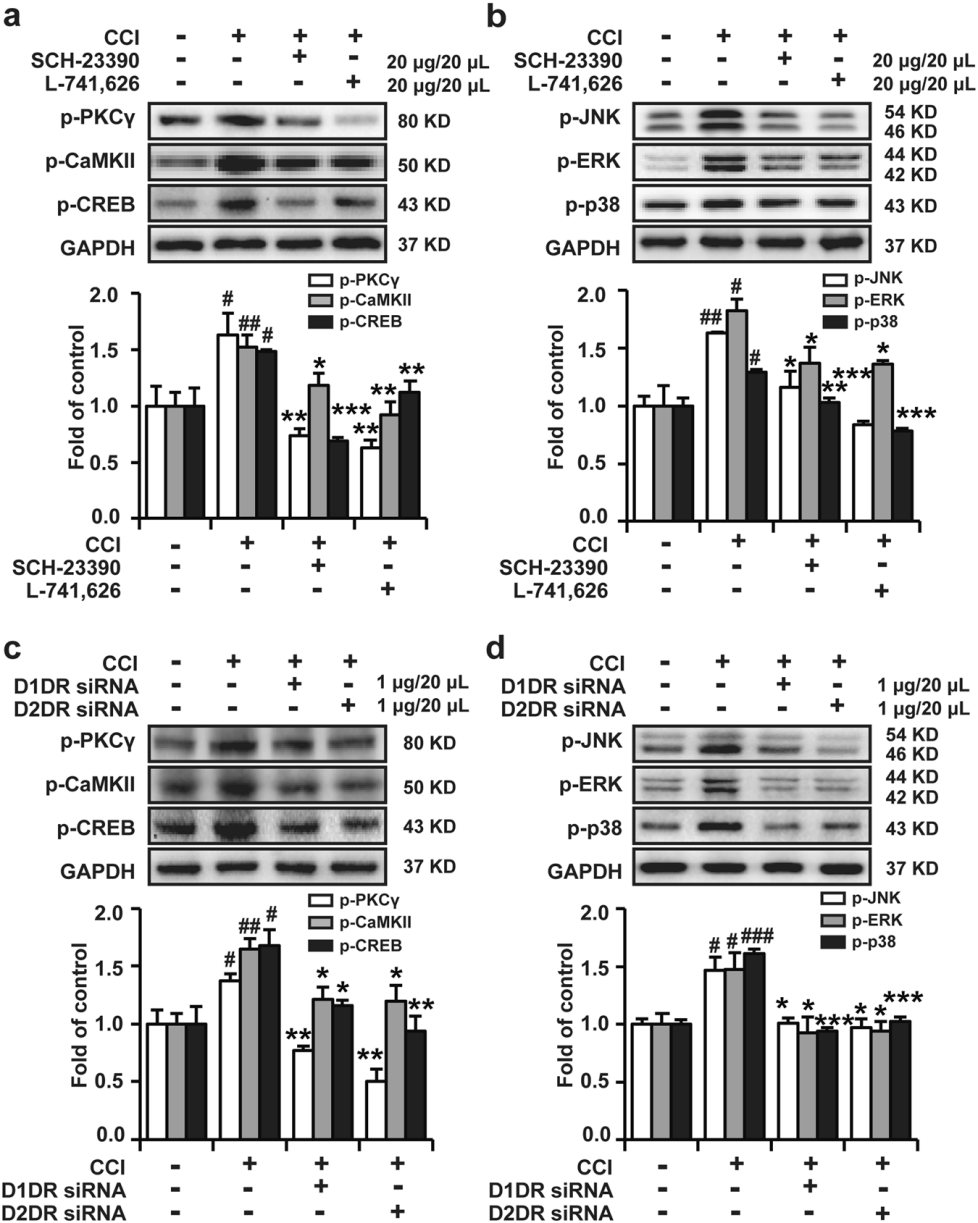
Antagonizing spinal D1DR and D2DR restrained the activation of PKC γ, CaMKII, MAPK, and CREB in CCI rats. **a**, **b** Western blotting showing the phosphorylation levels of PKC γ, CaMKII, CREB, JNK, p38, and ERK in the spinal cord after intrathecal administration of SCH-23390 (20 μg/20 μL, i.t.) and L-741,626 (20 μg/20 μL, i.t.). **c**, **d** Western blotting showing the expression of p-PKC γ, p-CaMKII, p-CREB, p-p38, p-ERK, and p-JNK in the spinal cord after intrathecal injection of D1DR and D2DR siRNA (1 μg/20 μL, i.t.) (*n* = 4; ^###^*P* < 0.001 vs the control group; *P < 0.05, **P < 0.01, ***P < 0.001 vs the CCI group).

D1–D2DR forms a heteromer that in turn activates Gαq-PLC-IP3-mediated calcium mobilization. An increase in the calcium concentration could lead to the activation of PKC γ and CaMKII, trigger the activation of MAPK and CREB phosphorylation, and promote gene expression^17^. Our results confirmed that p-PKC γ, p-CaMKII, p-MAPK, and p-CREB expression was upregulated in CCI rats, while SCH-23390 (20 μg/20 μL, i.t.) and L-741,626 (20 μg/20 μL, i.t.) significantly downregulated phosphorylated PKC γ, CaMKII, MAPK, and CREB expression (Fig. 4a, b). Furthermore, D1DR siRNA and D2DR siRNA (1 μg/20 μL, i.t.) dramatically suppressed the upregulation of spinal p-PKC γ, p-CaMKII, p-CREB (Fig. 4c), p-p38, p-ERK, and p-JNK (Fig. 4d) expression in CCI rats.

### The antinociceptive effects of *l*-CDL were mediated by the D1–D2DR complex in CCI rats

**Fig. 5:**
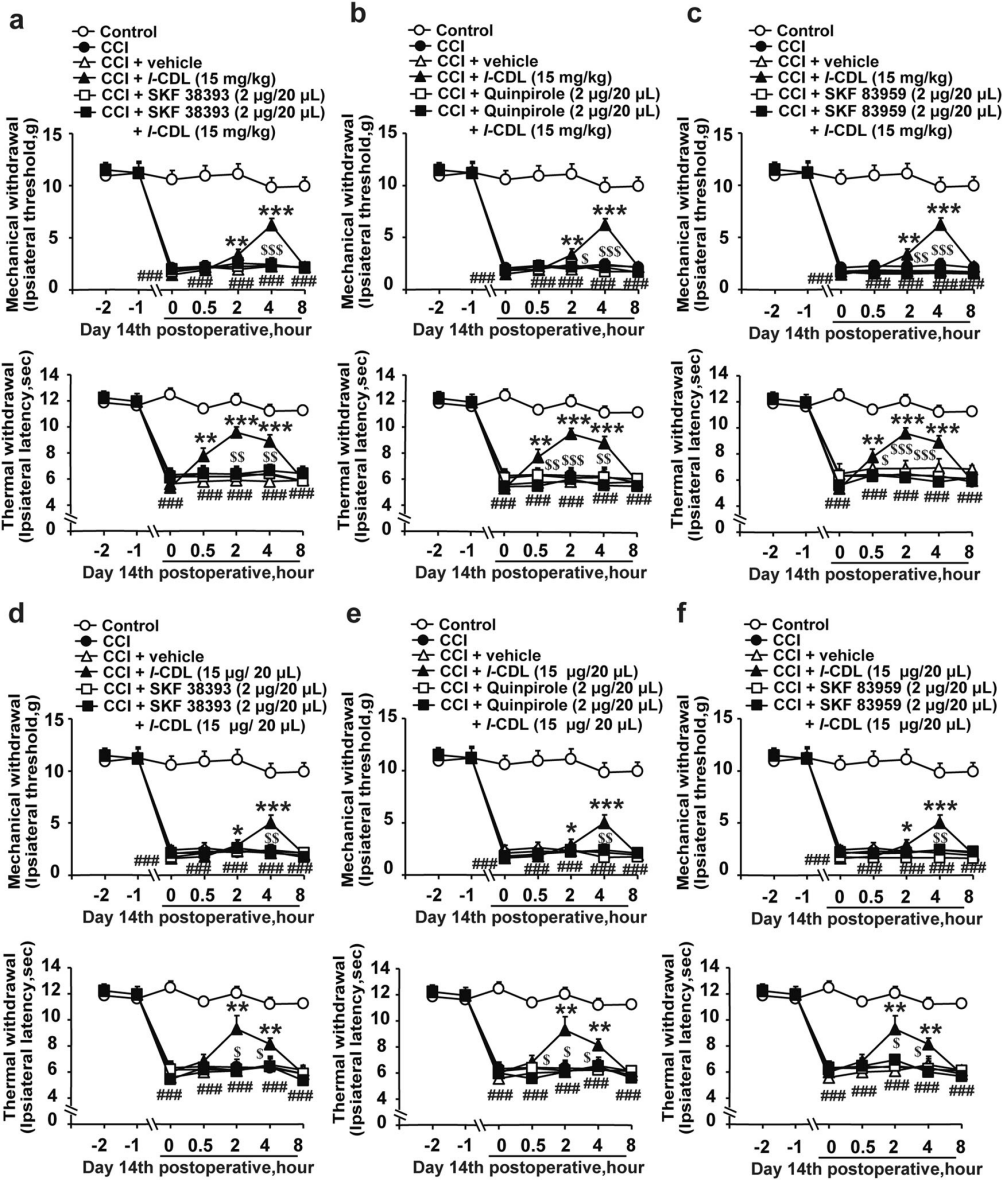
The antinociceptive effects of *l*-CDL were mediated by the D1–D2DR complex in CCI rats. **a**–**c** The time course of the MWTs and TWLs of CCI rats after coadministration of the D1DR agonist SKF 38393 (2 μg/20 μL, i.t.), D2DR agonist quinpirole (2 μg/20 μL, i.t.), and D1–D2DR heteromer agonist SKF 83959 (2 μg/20 μL, i.t.) with *l*-CDL (15 mg/kg, p.o.). **d**–**f** The time course of the MWTs and TWLs of CCI rats after coadministration of the D1DR agonist SKF 38393 (2 μg/20 μL, i.t.), D2DR agonist quinpirole (2 μg/20 μL, i.t.), and D1–D2DR heteromer agonist SKF 83959 (2 μg/20 μL, i.t.) with *l*-CDL (15 μg/20 μL, i.t.) (*n* = 6; ^###^*P* < 0.001 vs the control group; *P < 0.05, **P < 0. 01, ***P < 0.001 vs the CCI group; ^$^*P* < 0.05, ^$$^*P* < 0. 01, ^$$$^*P* < 0.001 vs the *l*-CDL group).

In our previous study, *levo*-corydalmine (*l*-CDL) was found to significantly attenuate neuropathic pain in CCI rats^31,32^. Moreover, our previous research showed that *l*-CDL exhibits micromolar affinity for both D1DR and D2DR with half maximal inhibitory concentrations (IC50) of 0.20 μM and 0.86 μM, respectively^33^. D1DR, D2DR, and D1–D2DR heterodimer agonists were used to investigate whether the *l*-CDL-induced analgesic effect is mediated by the D1–D2DR complex in the spinal cord. The antinociceptive effects of intragastric administration of *l*-CDL (15 mg/kg, p.o.) (Fig. 5a–c) and intrathecal administration of *l*-CDL (15 μg/20 μL, i.t.) (Fig. 5d–f) were abolished by SKF 38393, quinpirole and SKF 83959 but not by SKF 83822 (2 μg/20 μL, i.t., 15 min before *l*-CDL treatment) (Fig. s2a, b), indicating that *l-*CDL obviously relieved CCI-induced neuropathic pain by suppressing the D1–D2DR complex in the spinal cord.

### *l*-CDL suppressed dopamine D1–D2DR complex formation and decreased the intracellular calcium concentration in spinal neurons

**Fig. 6:**
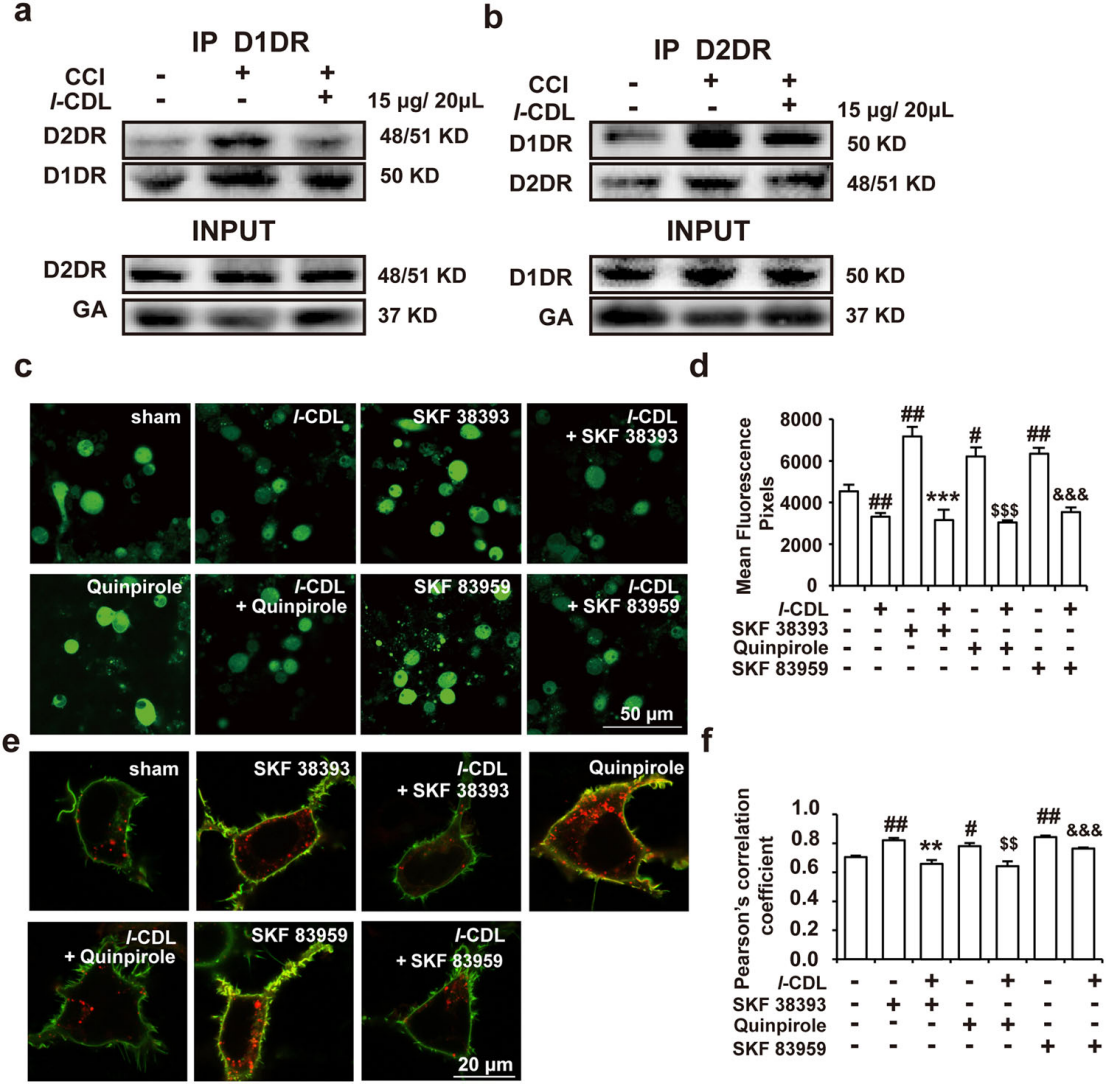
*l*-CDL suppressed dopamine D1–D2DR complex formation and decreased the intracellular calcium concentration in spinal neurons. **a**, **b** Coimmunoprecipitation of D1DR and D2DR in the spinal cords of CCI rats after intrathecal injection of *l-*CDL (15 μg/20 μL, i.t.). **c**, **d** Immunofluorescence showing the calcium concentration after administration of *l-*CDL (10 μM) and the effect of *l-*CDL (10 μM) on SKF 38393 (30 μM), quinpirole (30 μM), and SKF 83959 (30 μM)-induced augmentation of the calcium concentration in cultured spinal neurons. **e**, **f** Immunofluorescence showing the colocalization of D1DR and D2DR after administration of *l-*CDL (10 μM) and the effect of *l-*CDL on SKF 38393 (30 μM), quinpirole (30 μM), and SKF 83959 (30 μM)-induced increases in colocalization in the HEK293 cell membrane (*n* = 4; ^#^*P* < 0.05, ^##^*P* < 0.01 vs the control group; **P < 0.01, ***P < 0.001 vs the SKF 38393 group; ^$$^*P* < 0.01, ^$$$^*P* < 0.001 vs the quinpirole group; ^&&&^*P* < 0.001 vs the SKF 83959 group).

As shown in Fig. 6a, b, the coimmunoprecipitation results revealed that *l*-CDL inhibited the formation of the spinal D1–D2DR complex in CCI rats. Further in vitro experiments confirmed that *l*-CDL (10 μM) decreased calcium fluorescence in primary spinal neurons and that *l*-CDL effectively reduced the increase in the calcium concentration induced by SKF 38393 (30 μM), quinpirole (30 μM), and SKF 83959 (30 μM) (Fig. 6c, d). The increase in the calcium concentration in spinal neurons was also inhibited in the *l-*CDL alone-treated group compared with the sham group. In D1DR- and D2DR-transfected HEK293 cells, the results of fluorescence colocalization studies revealed that 10 μM *l*-CDL markedly inhibited SKF 38393-, quinpirole- and SKF 83959 (all 30 μM)-induced enhancement of colocalization of D1DR and D2DR (Fig. 6e, f).

### *l*-CDL-induced suppression of phosphorylated PKC γ, MAPK, CREB, and CaMKII was abolished by intrathecal administration of D1DR, D2DR, and D1–D2DR agonists

**Fig. 7:**
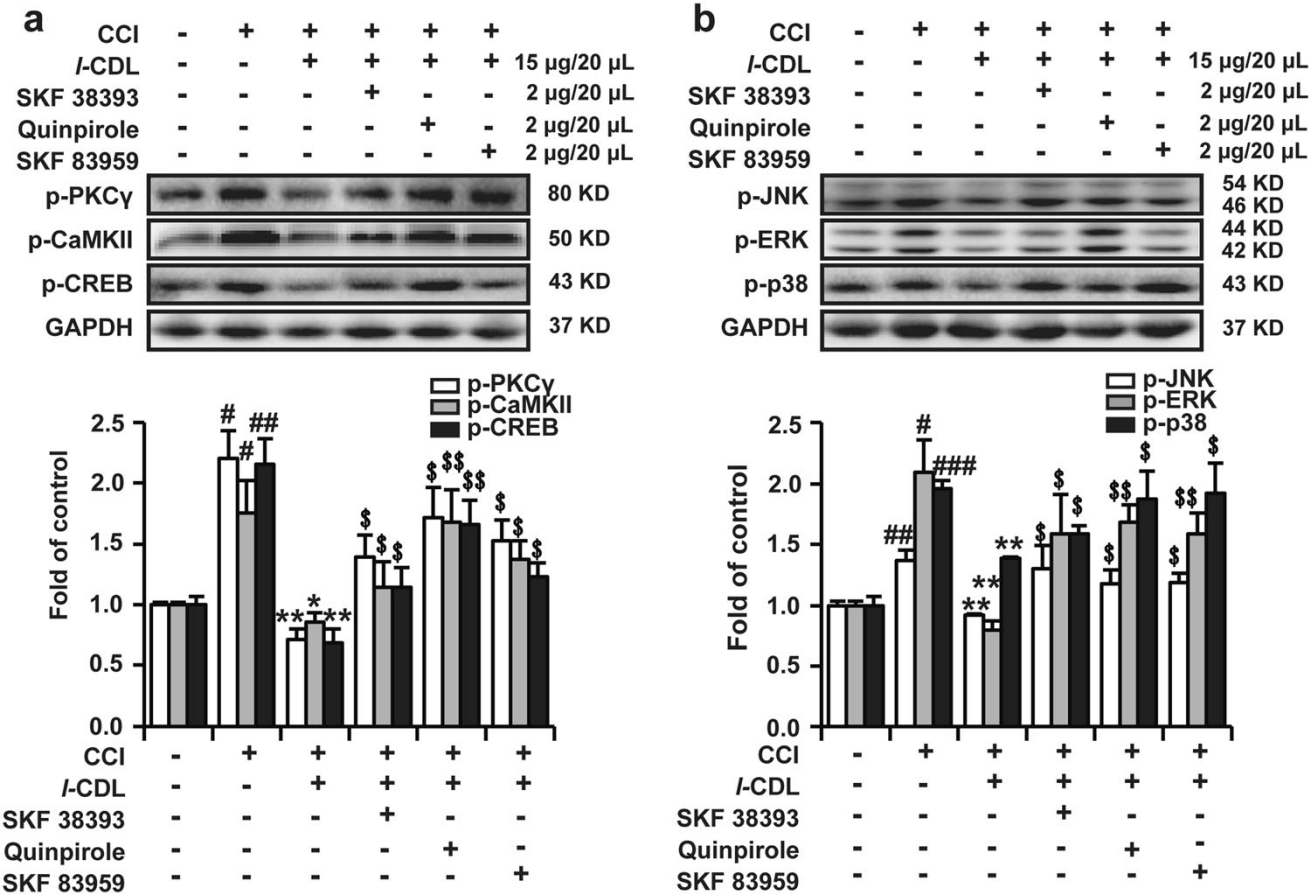
*l-*CDL-induced suppression of phosphorylated PKC γ, MAPK, CREB, and CaMKII was abolished by intrathecal administration of D1DR, D2DR, and D1–D2DR agonists. **a**, **b** Western blotting showing the phosphorylation levels of PKC γ, CaMKII, CREB, JNK, p38, and ERK in the spinal cord after coadministration of *l-*CDL (15 μg/20 μL) with SKF 38393, quinpirole, and SKF 83959 (*n* = 4; ^#^*P* < 0.5, ^##^*P* < 0.01 vs the control group; *P < 0.05, **P < 0.01 vs the CCI group; ^$^*P* < 0.05, ^$$^*P* < 0.01 vs the *l*-CDL group).

As indicated in Fig. 7a, b, the upregulation of p-PKC γ, p-MAPK, p-CREB, and p-CaMKII expression was effectively inhibited in *l*-CDL (15 μg/20 μL, i.t.)-treated group compared with the CCI group. Intrathecal administration of SKF 38393, quinpirole and SKF 83959 (2 μg/20 μL, i.t.) abolished *l*-CDL (15 μg/20 μL, i.t.)-induced suppression of phosphorylated PKC γ, CREB, CaMKII (Fig. 7a), JNK, ERK, and p38 expression (Fig. 7b) in the spinal cord.

## Discussion

**Fig. 8:**
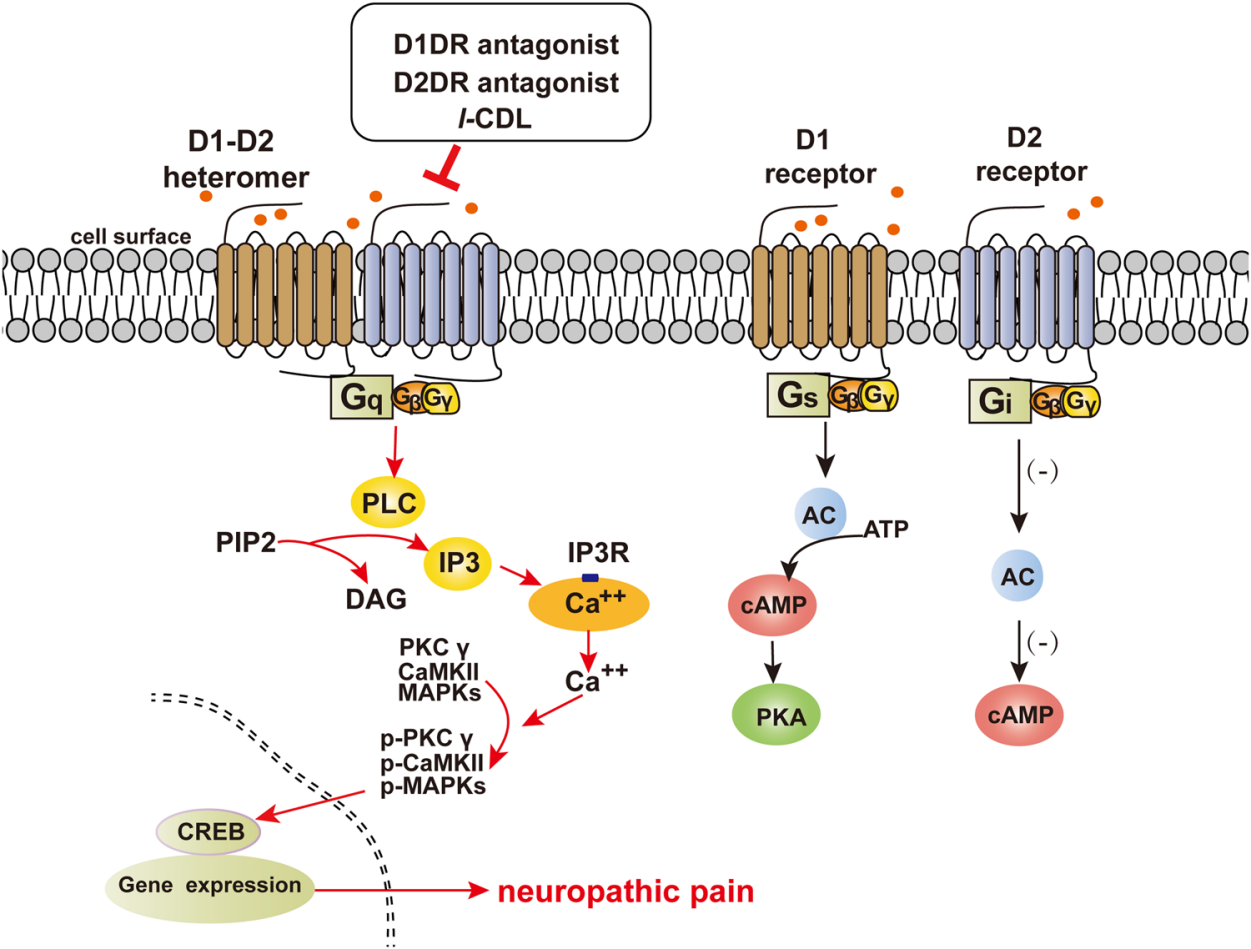
Proposed mechanisms of the D1–D2DR heteromer-mediated signaling pathway in neuropathic pain. Activation of the D1–D2DR heteromer leads to intracellular calcium mobilization from IP3 receptor-sensitive stores through a cascade of events involving rapid translocation of Gαq to the plasma membrane and activation of PLC. The increase in the calcium concentration leads to the promotion of PKC γ, CaMKII, MAPK, and CREB activation, which could increase the excitability of spinal neurons to promote the development of chronic neuropathic pain. *l-*CDL inhibits the formation of the D1–D2DR complex and downstream PKC γ, CaMKII, MAPK, and CREB signaling to alleviate CCI-induced neuropathic pain.

This research illustrated that suppressing the D1–D2DR complex could reduce neuronal excitability in the spinal cord via the regulation of calcium influx, thereby leading to the inhibition of CaMKII, PKC γ, and MAPK signaling to relieve neuropathic pain in CCI model rats. Our results showed that the natural analgesic compound *l*-CDL could inhibit the spinal D1–D2DR complex to reduce neuronal hyperexcitability and thus attenuate mechanical hyperalgesia and thermal allodynia in CCI rats (Fig. 8).

Neuropathic pain usually manifests as burning, shooting or stabbing pain^34^. After peripheral nociceptors are activated by a noxious stimulus, primary afferent nerves carry pain sensation from the DRG to the spinal cord, where the pain sensation is integrated and ultimately transmitted to the brain. The mechanisms driving neuropathic pain have been widely explored but are not fully understood.

Activation or antagonization of D1DR and D2DR has been reported to inhibit the development of pain. Intrathecal injection of the D2DR agonist quinpirole has been reported to produce short-term inhibition of the responses to cold and tactile stimuli in rats with chronic constriction injury of one sciatic nerve^35^. Intrathecal administration of the dopamine agonist apomorphine was reported to be unable to alleviate tail flick latency but to attenuate hot plate and acetic acid writhing responses^36^, while other studies have suggested that intrathecal administration of dopamine results in increases in tail-flick latency and mechanical allodynia, which can be alleviated by a D2DR antagonist but not a D1DR antagonist^37,38^. Furthermore, intrathecal administration of a D2DR agonist and a D1DR antagonist was reported to attenuate carrageenan-induced hyperalgesia^39^. However, it has been reported that upregulation of Krüppel-like factor 15 (KLF15) expression contributes to neuropathic pain partly by promoting the expression of D2DR^40^. In our research, we found that the expression of D1DR and D2DR was upregulated in the spinal cords of CCI rats, as shown in Fig. 1e, f, which also suggested that upregulated D1DR and D2DR expression might be involved in promoting neuropathic pain. In addition, it has been reported that the antinociceptive effects of the natural compound dehydrocorybulbine (DHCB) can be reversed by a D2DR agonist, as determined by the tail-flick assay, and that DHCB cannot alleviate pain in D2DR KO mice^41^. A clinical drug, rotundine, was also found to alleviate neuropathic pain through agonism of D1DR and antagonism of D2DR^42^. The different effects of dopamine receptor agonists and antagonists on pain observed in these studies might have been due to the use of different models and methods for measuring pain, but the main problem might be that the mechanisms by which D1DR and D2DR are involved in regulating pain still need to be explored.

D1DR and D2DR are traditional GPCRs that are known to exist as oligomeric complexes. It is well documented that D1DR and D2DR can form a novel and pharmacologically distinct receptor complex^16,17^, and D1DR and D2DR subtype-specific agonists (as well as D1DR and D2DR antagonists) activate (or inhibit) the formation of D1–D2DR heteromers^15^. In our research, the formation of the spinal D1–D2DR complex in the spinal cords of CCI rats was confirmed, and D1DR/D2DR antagonists were found to reduce the formation of the complex to alleviate CCI-induced neuropathic pain. The analgesic effect of D1DR and D2DR antagonists was reversed by coadministration of D1DR, D2DR, and D1–D2DR complex agonists, which further suggested that D1DR and D2DR antagonists inhibited neuropathic pain by suppressing the D1–D2DR complex.

The role of supraspinal descending modulation of dopamine in chronic pain has been demonstrated. It has been reported that A11 dopaminergic neurons send direct inhibitory projections to the spinal dorsal horn^43,44^ and that D1DR antagonists and D2DR agonists exert antinociceptive effects on the dopaminergic pathway from the A11 nucleus to the spinal trigeminal nucleus caudalis or spinal dorsal horn^45^. Intrastriatal administration of a D2DR agonist attenuates neuropathic hypersensitivity in rats^46^. However, antagonism of supraspinal D2DR by *l-*tetrahydropalmatine (THP) was found to alleviate pain^9^. A D2DR antagonist was also found to alleviate migraine^47,48^. In addition, it is generally assumed that D1DR and D2DR play opposite roles in pain modulation^7^. It has been reported that the two types of dopamine receptors are differentially expressed in different neurons^49,50^, causing them to have different roles in pain modulation. However, it has also been reported that D1DR and D2DR are coexpressed in >90% of cultured striatal neurons and can form a D1–D2DR complex^17^. Many other researchers have indicated that D1DR and D2DR are colocalized in neurons^51,52^. We wondered whether the degree of D1DR and D2DR colocalization is associated with pain in the brain. Our research indicated that spinal D1DR and D2DR form complexes to promote chronic pain and that D1DR and D2DR antagonists can decrease the D1–D2DR complex to alleviate neuropathic pain.

It has been reported that the D1–D2DR complex couples with Gαq to form a Gαq-coupled D1–D2DR heteromer, which has been reported to activate the PLC-IP3-mediated calcium pathway^53^. Changes in the intracellular concentration of calcium in neurons are involved in regulating neuronal excitability, which may result in the facilitation of neuropathic pain^54^. Numerous previous studies have suggested that PLC cleaves PIP2 into DAG and IP3, thus leading to the release of intracellular stored calcium ions, which could activate PKC γ and CaMKII. The activation of PKC γ and CaMKII further activates the MAPK signaling cascade, thus triggering the activation of the transcription factor CREB^55,56^. Inhibition of PKC γ, CaMKII, MAPK, and CREB can suppress the generation and maintenance of neuropathic pain^57^.

*l*-CDL, a natural active analgesic ingredient from the traditional Chinese medicine *Corydalis yanhusuo* W.T. Wang, has been proven to be effective in relieving chronic pain^58,59^. In our previous research, *l-*CDL was found to inhibit NMDA receptors and the mGlu1/5 receptor to suppress the activation of spinal neurons and thus relieve chronic pain^59^. *l-*CDL exhibits micromolar affinity for both D1DR and D2DR with half maximal inhibitory concentrations (IC50) of 0.20 μM and 0.86 μM, respectively^33^. Dopamine receptors have also been reported to regulate NMDA function in neurons, and our results indicated that D1DR and D2DR could activate NMDA to increase the excitability of spinal neurons and thus promote chronic pain in a Gαq-dependent manner^60^. These results suggest that D1DR and D2DR might form a D1–D2DR complex, leading to the activation of the Gαq protein in the spinal cord and thus activation of NMDA receptors in chronic pain.

In conclusion, our study suggests that dopamine D1DR and D2DR form a complex in the spinal cord to promote chronic neuropathic pain by activating the Gαq protein and downstream PKC γ, CaMKII, MAPK, and CREB signaling to increase the excitability of spinal neurons and thus may be drug targets for neuropathic pain. *l*-CDL targets D1DR and D2DR to reduce the formation of the dopamine D1–D2DR complex to relieve CCI-induced neuropathic pain.

## Supplementary information

Supplementary Material
