# Supplementary material for: The dopamine D1–D2DR complex in the rat spinal cord promotes neuropathic pain by increasing neuronal excitability after chronic constriction injury
Source: Exp Mol Med. 2021 Feb 9;53(2):235–49. doi: 10.1038/s12276-021-00563-5 (PMC8080784; doi:10.1038/s12276-021-00563-5)
Supplement: Supplementary file 1 — Supplementary Material [file 12276_2021_563_MOESM1_ESM.doc]

## Supplementary Material

## Supplementary Figures


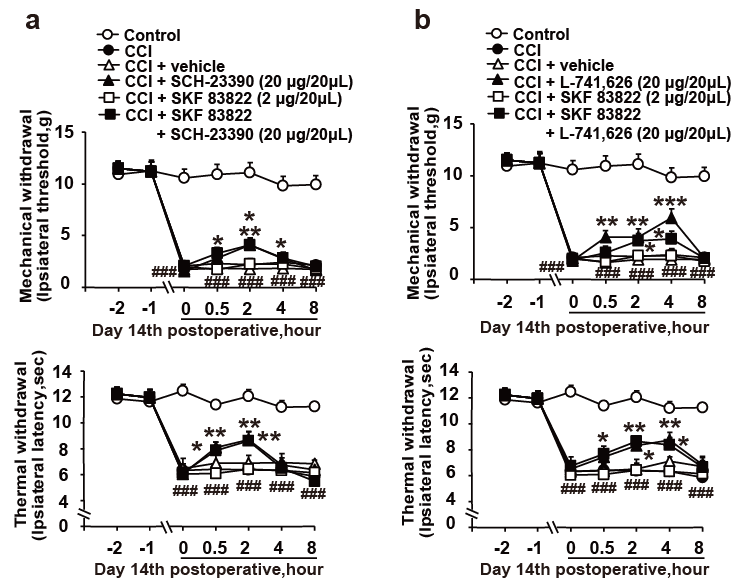


**Fig. s1** Intrathecal administrated SKF-83822 could not affect the antinociception of D1DR, D2DR antagonists in CCI rats. Behavioral results showed that D1DR agonist SKF-83822 (robustly stimulated AC, 2 μg/ 20 μL, i.t.) had no effect on D1DR antagonist SCH-23390 (20 μg/ 20 μL, i.t.)) (a) and D2DR antagonists L-741,626 (20 μg/ 20 μL, i.t.) (b) induced antinociception (n = 6, ###*P* < 0.001, compared with control group; **P* < 0.05, ***P* < 0. 01, ****P* < 0.001, compared with CCI group).


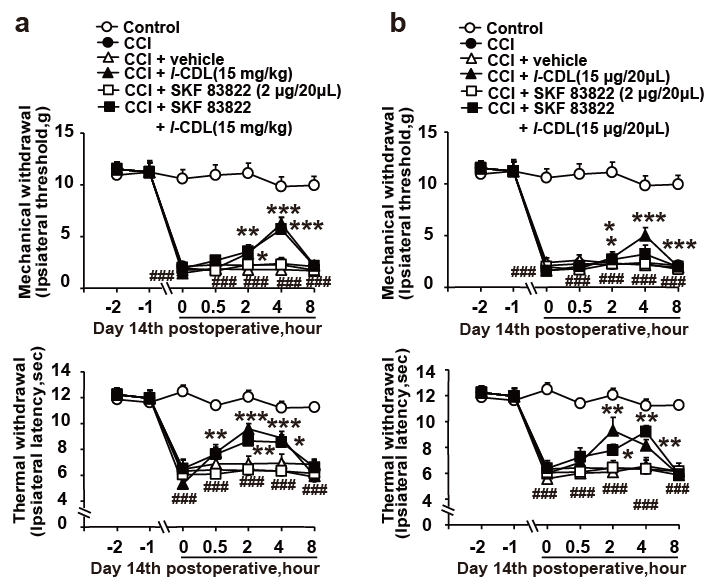


**Fig. s2** Intrathecal administrated SKF-83822 had no effect on the antinociception of *l*-CDL in CCI rats. Intragastric administration of *l*-CDL (15mg/kg, p.o.) (a) and intrathecal administration of *l*-CDL (20 μg/20μL, i.t.) (b) both reduced CCI-induced neuropathic pain, while SKF-83822 (2μg/ 20 μL, i.t.) did not affect the analgesic effect of *l*-CDL (n = 6, ###*P* < 0.001, compared with control group; **P* < 0.05, ***P* < 0. 01, ****P* < 0.001, compared with CCI group).
